# S1‐Leitlinie für bildgebende Diagnostik von Hauterkrankungen

**DOI:** 10.1111/ddg.15883_g

**Published:** 2025-12-11

**Authors:** Maximilian Deußing, Sandra Schuh, Janis Thamm, Deborah Winkler, Simon Schneider, Teresa Nau, Ulf Darsow, Viktor Schnabel, Martina Ulrich, Lynhda Nguyen, Denis Frenzel, Chiara Fischer, Cristel Ruini, Vasilis Ntziachristos, Martin Kaatz, Hjalmar Kurzen, Bernd Kardorff, Rudolf Herbst, Sonja Grunewald, Elke Sattler, Julia Welzel, Daniela Hartmann

**Affiliations:** ^1^ Klinik und Poliklinik für Dermatologie und Allergologie Ludwig‐Maximilians‐Universität München München Deutschland; ^2^ Klinik für Dermatologie Allergologie und Lasermedizin München Klinik München Deutschland; ^3^ Klinik für Dermatologie und Allergologie Universitätsklinikum Augsburg Augsburg Deutschland; ^4^ Klinik und Poliklinik für Dermatologie und Allergologie am Biederstein Technische Universität München München Deutschland; ^5^ Klinik und Poliklinik für Dermatologie Venerologie und Allergologie Universitätsklinikum Leipzig Leipzig Deutschland; ^6^ Dermatologie am Regierungsviertel Berlin Deutschland; ^7^ Klinik und Poliklinik für Dermatologie und Venerologie Universitätsklinikum Hamburg‐Eppendorf (UKE) Hamburg Deutschland; ^8^ Praxisklinik Starnberg Starnberg Deutschland; ^9^ Lehrstuhl für Biologische Bildgebung Technische Universität München München Deutschland; ^10^ Klinik und Poliklinik für Dermatologie Sapienza Universität Rom Rom Italien; ^11^ Institute of Biological and Medical Imaging Helmholtz Zentrum München Neuherberg Deutschland Chair of Biological Imaging Central Institute for Translational Cancer Research (TranslaTUM) School of Medicine Technische Universität München München Deutschland; ^12^ Hautklinik DRK Krankenhaus Chemnitz‐Rabenstein Chemnitz Deutschland; ^13^ Haut‐ und Laserzentrum Freising Freising Deutschland; ^14^ Haut‐ Allergie‐ Venen‐ und Laserpraxen Prof. Dr. Kardorff medermis clinics Mönchengladbach Deutschland; ^15^ Klinik für Hautkrankheiten und Allergologie Helios Klinikum Erfurt Erfurt Deutschland

**Keywords:** Basalzellkarzinom, malignes Melanom, nichtinvasive Bildgebung, physikalische Diagnostik, Plattenepithelkarzinom, Basal cell carcinoma, malignant melanoma, non‐invasive imaging, physical diagnosis, squamous cell carcinoma

## Abstract

Nichtinvasive Bildgebungstechniken erlauben schnell und schmerzfrei diagnostische Blicke in die Haut, je nach angewandter Methode mit unterschiedlich hoher Auflösung und Eindringtiefe.

Schon seit Jahrzehnten aus dem Alltag nicht wegzudenken sind die etablierten Untersuchungsmethoden wie die Dermatoskopie und die hochauflösende Sonographie der Haut und Subkutis. Weitere innovative Verfahren wie die optische Kohärenztomographie (OCT), die konfokale Lasermikroskopie (KLM) und die *Line‐field* konfokale OCT (*Line‐field confocal OCT*; LC‐OCT), die die Untersuchung der Haut mit sehr hoher Auflösung erlauben, haben Einzug in die klinische Routine gehalten. Die Multiphotonentomographie sowie die optoakustische Bildgebung gelten ebenfalls als vielversprechende neuere Verfahren.

Neben den nichtinvasiven Verfahren, die in vivo direkt am Patienten zum Einsatz kommen, können die KLM und die LC‐OCT auch ex vivo an exzidiertem Gewebe zur Schnellschnittdiagnostik angewendet werden.

Die bei allen Bildgebungsmethoden generierten Daten eignen sich hervorragend für die Anwendung KI‐basierter Algorithmen zur Erhöhung der diagnostischen Treffsicherheit und Unterstützung des erfahrenen Anwenders.

Alle genannten Verfahren haben ihre Stärken, Limitationen und bevorzugte Indikationen. Im Folgenden wird ein Überblick über die verschiedenen Geräte und Techniken gegeben und für jede Methode die Funktionsweise und die aktuelle Studienlage zu Indikationen und Grenzen des jeweiligen Verfahrens dargelegt.

## INFORMATIONEN ZU DIESER LEITLINIE

Diese Version der Leitlinie ist eine Kurzfassung der vollständigen Leitlinie, die als Online‐Supplement und unter www.awmf.org frei verfügbar ist. Eine vollständige Liste der Referenzen und eine Übersichtstabelle, die den Empfehlungen und Statements dieser Leitlinie zugrunde liegen sowie die Interessenkonflikte der beteiligten Autoren sind in der Langversion beziehungsweise im Leitlinienreport enthalten.

Die vorliegende erste deutsche Leitlinie Bildgebende Diagnostik von Hauterkrankungen wurde unter der Federführung der Deutschen Dermatologischen Gesellschaft (DDG) und der Arbeitsgemeinschaft für Physikalische Diagnostik in der Dermatologie (ApDD) entwickelt und beruht auf systematischen Literaturrecherchen und dem Konsens der Expertengruppe. Die Leitlinie beinhaltet Empfehlungen zu den Bereichen Technik, Indikationen, Evidenz und Limitationen der verschiedenen Bildgebungsmethoden.

Die Medizin unterliegt einem fortwährenden Entwicklungsprozess, sodass alle Angaben, insbesondere zu diagnostischen und therapeutischen Verfahren, immer nur dem Wissensstand zur Zeit der Drucklegung der Leitlinien entsprechen können. Im Hinblick auf die angegebenen Empfehlungen zur Therapie und der Auswahl sowie Dosierung von Medikamenten wurde die größte Sorgfalt beachtet. Gleichwohl werden die Benutzer angewiesen, die Beipackzettel und Fachinformationen des Herstellers zur Kontrolle heranzuziehen und im Zweifelsfall einen Spezialisten zu konsultieren. Der Benutzer selbst bleibt verantwortlich für jede diagnostische und therapeutische Applikation, Medikation und Dosierung. In dieser Leitlinie sind eingetragene Warenzeichen (geschützte Warennamen) nicht besonders bekannt gemacht. Es kann auch aus dem Fehlen eines entsprechenden Hinweises nicht geschlossen werden, dass es sich um einen kostenlosen Warennamen handelt.

## KONFOKALE LASERMIKROSKOPIE (KLM)

### Technik

Die konfokale Lasermikroskopie (KLM) ist vor allem zur nichtinvasiven Diagnostik melanozytärer und epithelialer Hauttumoren geeignet.^1^ Hierbei wird Laserlicht einer ausgewählten Wellenlänge auf eine Ebene innerhalb der Haut fokussiert, wo das Licht an Grenzflächen mit hohem Brechungsindex (Keratin, Melanin und Kollagen) reflektiert und dann auf einen Detektor geleitet wird. Eine vorgeschaltete Lochblende ermöglicht, dass ausschließlich Signale aus der vorab definierten horizontalen Ebene zur Bildgebung herangezogen werden. Während diese Vorgehensweise einerseits die hochauflösende Darstellung oberflächennaher Veränderungen mit mikroskopischer Auflösung von 1 bis 3 µm in horizontaler Schnittführung erlaubt, bedingt sie gleicherweise die Limitierung der Eindringtiefe in die Haut.

### Geräte

Für die konfokale Lasermikroskopie werden Geräte mit einem oder mehreren Lasern als Lichtquelle eingesetzt, die sowohl zur In‐vivo‐ als auch *Ex‐vivo*‐Untersuchung der Haut herangezogen werden können. Die Laserenergie auf Gewebsebene beträgt weniger als 30 mW, daher besteht keine Gefahr für das zu untersuchende Gewebe oder das menschliche Auge (Laserklasse I).

### Indikationen

#### Melanozytäre Läsionen

Durch den hohen Brechungsindex von Melanin lassen sich insbesondere melanozytäre Läsionen sehr gut darstellen. Folglich wurden bildmorphologische Charakteristika zur Unterscheidung von benignen und suspekten Läsionen erarbeitet.^2^, ^3^

**ABBILDUNG 1:**
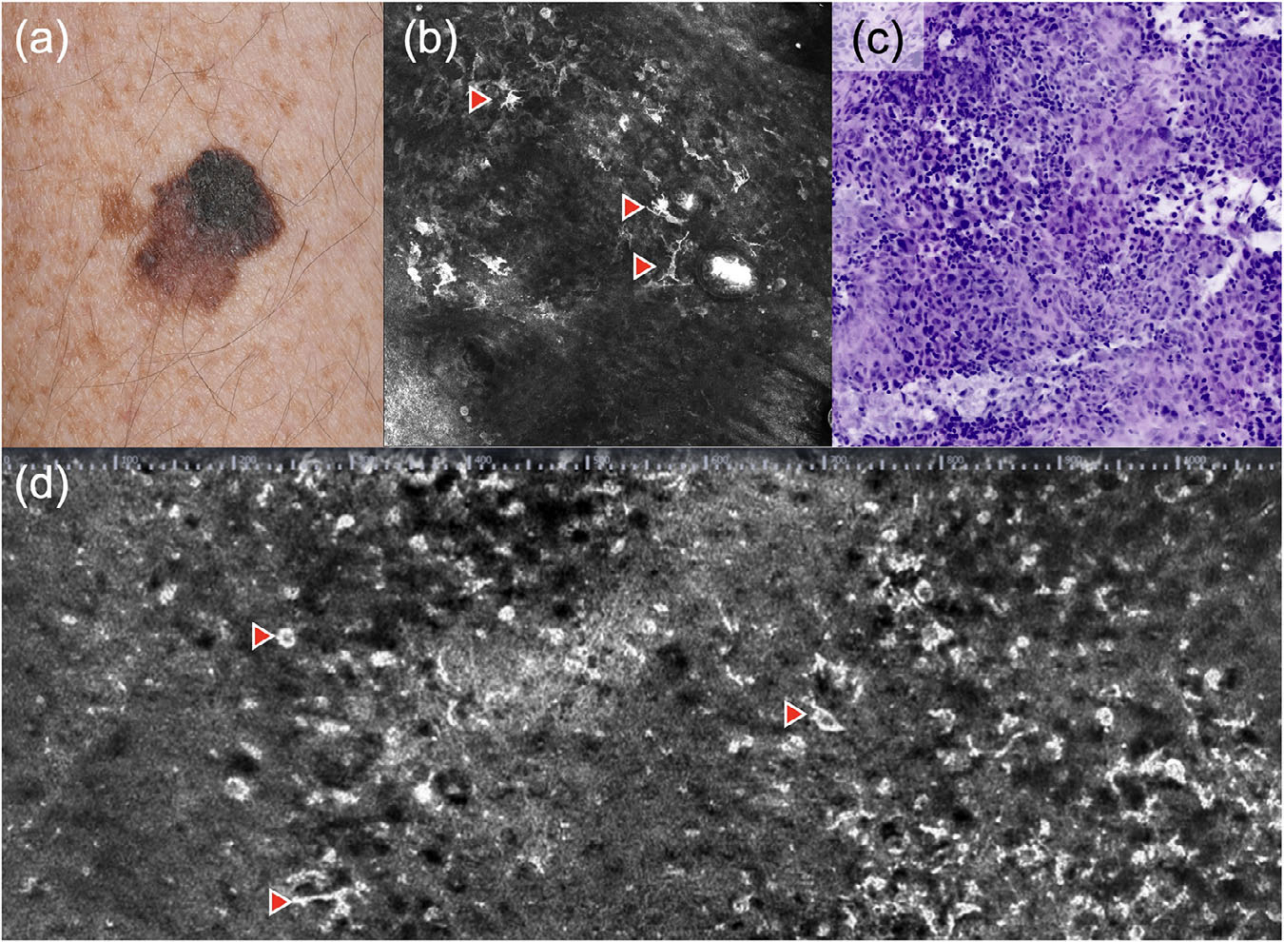
Darstellung eines superfiziell spreitenden Melanoms. (a) Klinisches Bild; (b) horizontale in vivo konfokale Laserscanmikroskopie (VivaScope 1500, 750 µm × 750 µm, VivaScope GmbH, München, Deutschland); (c) ex vivo konfokale Laserscanmikroskopie eines nodulären Melanoms (VivaScope 2500M‐G4, VivaScope GmbH, München, Deutschland) mit Darstellung von dermalen Nestern atypischer Melanozyten im digitalen HE‐Modus; (d) vertikale *Line‐field* konfokale optische Kohärenztomographie (DeepLive, DAMAE Medical, Paris, Frankreich) mit Darstellung pagetoider und dendritischer Zellen (Pfeile).

In diesem Zusammenhang gelten eine Aufhebung der normalen Epidermisarchitektur (atypisches Honigwabenmuster), der normalen dermoepidermalen Junktionsstruktur (DEJ; abrupte DEJ), eine fehlende Abgrenzbarkeit der dermalen Papillen (non‐edged papillae), das Vorhandensein von großen, hochrefraktilen Zellen mit prominenten Nuklei in höheren Epidermislagen (runde und dendritische pagetoide Zellen [Abbildung 1]), irreguläre Nester atypischer Melanozyten (dichte und schüttere Nester, cerebriforme Nester) sowie kleine, hochrefraktile Partikel (inflammatorische Partikel) als wichtigste Kriterien für eine maligne Transformation. Bei amelanotischen Melanomen ist die Diagnose mittels KLM erschwert, allerdings gelten asymmetrische, pigmentierte Follikel, ≥ 3 atypische Zellen in fünf Feldern, und fokale follikuläre Ausdehnung der atypischen Zellen bei der DEJ als Schlüsselkriterien für die Unterscheidung von anderen Hauttumoren.^4^

Zahlreiche Studien zeigen, dass die KLM zur Verbesserung der Spezifität in der Melanomdiagnostik im Vergleich zur Dermatoskopie allein führt, insbesondere bei unklaren Läsionen und auch in randomisierten, kontrollierten Studien.^5^, ^6^, ^7^, ^8^ Letztendlich kommt es durch die Anwendung der KLM zur Reduktion unnötiger Exzisionen und zur Früherkennung auch dünner Melanome. Zudem kann die KLM die *number needed to excise* (NNE) signifikant verringern,^8^, ^9^ und somit die Kosten für das Gesundheitssystem senken.^9^

#### Basalzellkarzinom

Die KLM eignet sich auch zur Untersuchung nichtmelanozytärer Hauttumoren.^10^, ^11^, ^12^, ^13^, ^14^, ^15^, ^16^

**ABBILDUNG 2:**
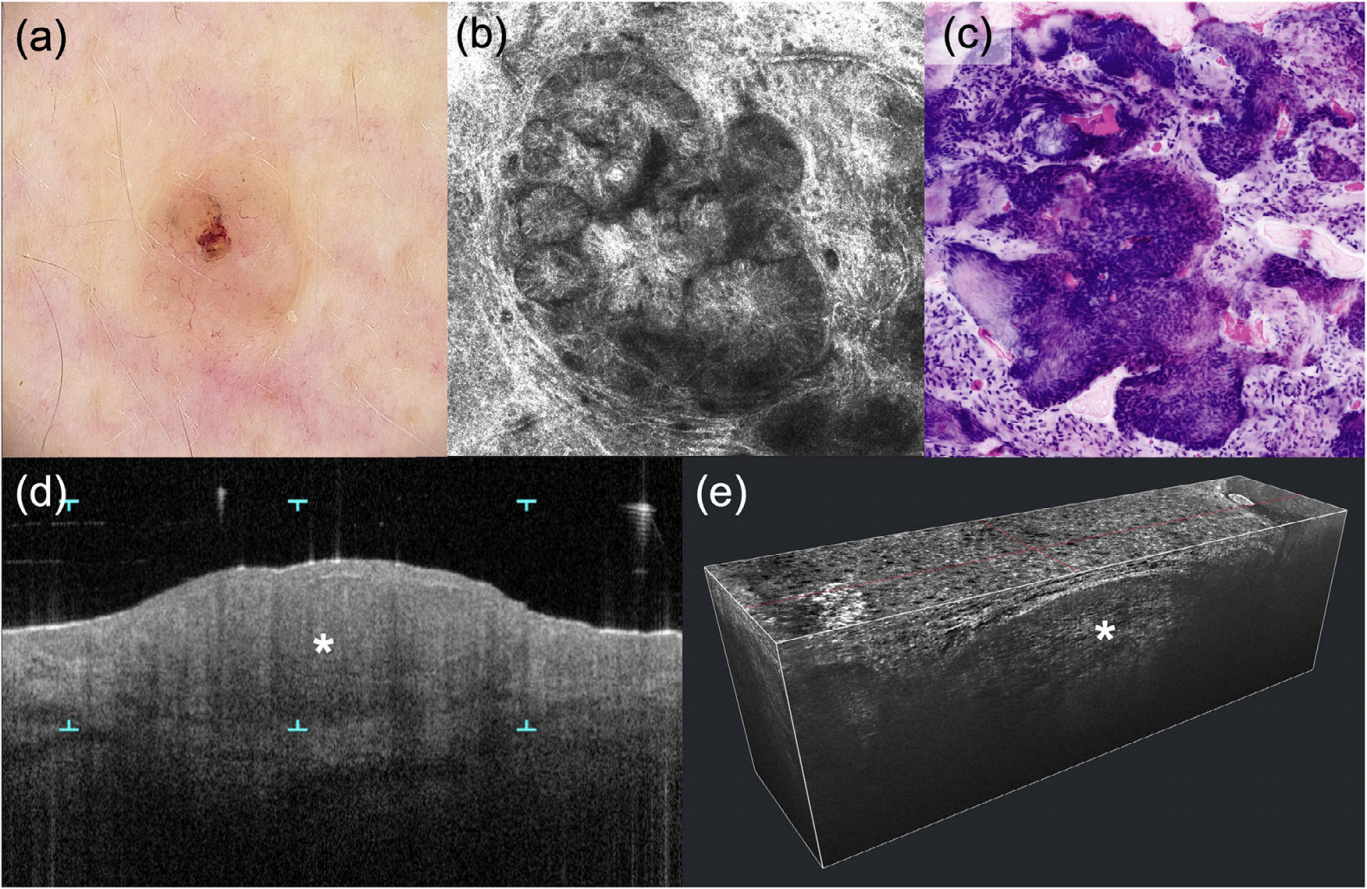
Solides Basalzellkarzinom. (a) Dermatoskopische Aufsicht (20‐fache Vergrößerung); (b) in vivo konfokale Laserscanmikroskopie (VivaScope 1500, 750 µm × 750 µm, VivaScope GmbH, München, Deutschland) mit Darstellung charakteristischer Tumorinseln; (c) ex vivo konfokale Laserscanmikroskopie (VivaScope 2500M‐G4, VivaScope GmbH, München, Deutschland) in digitaler H&E‐Färbung mit Darstellung von Tumorzellverbänden; (d) optische Kohärenztomographie (VivoSight, Michelson Diagnostics Ltd, Kent, UK) in vertikaler Schnittebene zur Darstellung des Tumorknotens (Stern); (e) *Line‐field* konfokale optische Kohärenztomographie (DeepLive, DAMAE Medical, Paris, Frankreich) in 3D‐Darstellung.

So wurden folgende fünf Hauptkriterien zur Diagnosestellung eines Basalzellkarzinoms beschrieben: elongierte, monomorphe Zellkerne, Polarisierung dieser Zellen entlang einer Achse, ausgeprägtes Entzündungsinfiltrat, vermehrte sowie dilatierte Gefäße und Verlust der epidermalen Honigwabenstruktur.^17^ Als charakteristisch gelten zudem Inseln von Tumorzellen mit peripherer Palisadenstellung in der Dermis, die sich vom Bindegewebe durch einen dunklen Spalt abgrenzen (Abbildung 2). Diese optische Spaltbildung entspricht histologisch der Ansammlung von Muzin.^18^ In einer großen Multicenter‐Studie konnte eine hohe Sensitivität der KLM von 100% und Spezifität von 88,5% in der Diagnostik des Basalzellkarzinoms gezeigt werden.^18^

#### Aktinische Keratosen und Plattenepithelkarzinom

In der KLM sind aktinische Keratosen durch einen Verlust der normalen Honigwabenstruktur mit Atypien und Pleomorphismus epidermaler Keratinozyten, Parakeratose, losgelöste Korneozyten im Stratum corneum und solarer Elastose sowie Blutgefäßdilatation gekennzeichnet.^19^, ^20^, ^21^, ^22^, ^23^

Morbus Bowen/plattenepitheliale Carcinomata in situ zeigen neben der atypischen Honigwabenstruktur Dyskeratosen und typischerweise glomeruläre Gefäße.^24^, ^25^

#### Infektiöse und parasitäre Dermatosen

Die nichtinvasive Bildgebung der Haut zeigt aufgrund der hohen Korrelation mit histopathologischen Merkmalen auch bei entzündlichen und infektiösen Hauterkrankungen vielversprechende Ergebnisse.^26^, ^27^

Bei infektiösen Erkrankungen wie Mykosen und parasitären Dermatosen eignet sich die KLM zur direkten Erregerdiagnostik. Gerade oberflächliche Mykosen wie die Tinea corporis, cruris und manuum und sogar bei der klinisch schwierig einzuordnenden Tinea incognita gelingt durch KLM häufig der Direktnachweis mykotischen Materials in der Epidermis.^28^, ^29^, ^30^, ^31^ Neben Trichophyten‐Infektionen wurden mittels KLM in befallenen Regionen auch Candida‐Pseudofilamente und Konidien,^26^ sowie Malassezia‐Spezies mit typischen „Spaghetti und Fleischbällchen“‐Äquivalenten nachgewiesen.^27^

Im Falle der Onychomykose ist die gute Darstellbarkeit des Mycels und/oder der Sporen selbst in tieferen Nagelbereichen gewährleistet, da die optische Beschaffenheit des Nagels eine höhere Eindringtiefe erlaubt. Hyphen und Sporen stellen sich als hell reflektierende Strukturen mit typischer Morphologie dar.^29^, ^32^ So erzielt die KLM in der Onychomykose‐Diagnostik einen hohen prädiktiven Wert und eine hohe Spezifität,^33^ und zeigt sich in vergleichenden Studien vielen konventionellen Nachweismethoden sogar überlegen.^29^, ^32^, ^34^

Auch parasitäre Milben wie *Sarcoptes scabiei* oder *Demodex folliculorum* sind eindeutig identifizierbar. Im Fall der Skabies ermöglicht die KLM zum Beispiel eine schnelle Diagnostik mit hoher Sensitivität und Spezifität.^35^, ^36^, ^37^, ^38^ Im Rahmen des Therapiemonitorings der Rosacea ermöglicht die KLM eine der Histologie überlegene Quantifizierung der Milbendichte^38^, ^39^ und bietet sich zudem zur Diagnostik anderer Demodex‐assoziierter Erkrankungen wie der Demodex‐Blepharitis an.^40^

#### Entzündliche Dermatosen

Die psoriasiforme Dermatitis ist stark mit einer Verdickung der Epidermis assoziiert, während die spongiotische Dermatitis die typischen KLM‐Merkmale einer epidermalen Spongiose und Bläschenbildung aufweisen. Prototypische Erkrankungen der psoriasiformen Dermatitis wie die Plaquepsoriasis und die seborrhoische Dermatitis können auf Basis ihrer morphologischen Unterschiede noch weiter differenziert werden.^41^, ^42^ Größere Untersuchungen zur Sensitivität und Spezifität liegen derzeit allerding noch nicht vor.

Zu den typischen Vertretern der spongiotischen Dermatitis zählten die irritative und die allergische Kontaktdermatitis. Beide können mittels KLM – durch Beurteilung der Reaktion des Stratum corneums, dem Vorliegen epithelialer Nekrosen und unterschiedlicher Kinetiken – voneinander abgegrenzt werden.^43^, ^44^, ^45^ Unklare Ergebnisse im Epikutantest lassen sich so experimentell ohne die Entnahme von Biopsien sicher zuordnen.^43^, ^46^, ^47^, ^48^

### Limitationen

Generell erfordert die KLM detaillierte histologische Kenntnisse der Histologie und Pathologie der Haut zur korrekten Interpretation der Bilder, sodass diese Technik in Form von Trainingskursen erlernt werden sollte. Die größte Limitation der KLM ist ihre geringe Eindringtiefe bis in das Stratum papillare der Dermis. So entgehen alle tieferen dermalen Veränderungen wie zum Beispiel bei nodulären Melanomen, knotigen Basalzellkarzinomen oder Pannikulitiden der konfokalen Diagnostik. Sie eignet sich nur für die Diagnostik von Erkrankungen und Tumoren, die in der Epidermis und oberen Dermis ihre charakteristischen Veränderungen zeigen. Ebenso können die relativ lange Messdauer und das kleine Messfeld zu Einschränkungen führen. Patienten müssen in der Lage sein, einige Minuten still zu halten. Stark gewölbte, eingesunkene, keratotische oder nässende Hautveränderungen sind schwierig zu messen, da Oberflächenveränderungen zu Artefakten und Signalschatten führen können.

## EX‐VIVO‐KONFOKALE LASERSCANMIKROSKOPIE (Ex‐vivo‐KLM)

### Technik

Die *Ex‐vivo*‐konfokale Laserscanmikroskopie (*Ex‐vivo*‐KLM) eignet sich insbesondere zur Untersuchung frischer Gewebeexzidate bei der Schnellschnittdiagnostik,^15^ und hat dabei weniger Limitationen bezüglich der Eindringtiefe, da die Proben mit der jeweiligen Schnittfläche aufgelegt werden.^49^

Die *Ex‐vivo*‐KLM erlaubt innerhalb von Minuten eine feingewebliche Untersuchung der Haut ohne die nachfolgende konventionelle histologische Untersuchung zu beeinträchtigen. Dabei werden die Proben nach Fluoreszenzfärbung analog zur In‐vivo‐KLM mittels Laserlicht untersucht,^51^ und Bilder über einen Reflexions‐ (RM) und/oder einen Fluoreszenzmodus (FM) generiert.^52^

Neben den unterschiedlichen Brechungsindizes zellulärer Kompartimente^53^ kann durch die Fluoreszenz ein zusätzlicher Kontrast erzeugt werden, der von der histologischen Struktur und den Eigenschaften des eingesetzten Fluorophors geprägt ist.

Es wurden bislang zahlreiche Fluoreszenzfarbstoffe beschrieben,^54^ allerdings hat sich in der Praxis Acridinorange aufgrund seines guten Kern‐Zytoplasma‐Kontrasts und seiner geringen Ausbleichung bewährt.^50^, ^55^ Aufgenommene Bilder können softwareseitig in Hämatoxylin‐Eosin (HE)‐Färbung ähnliche Abbildungen konvertiert werden.^56^

### Indikationsspektrum

#### Basalzellkarzinom

Die *Ex‐vivo‐*KLM ist hervorragend zur feingeweblichen Diagnostik von Basalzellkarzinomen geeignet. Die Darstellung der Tumorzellproliferate in der digitalen Färbung ist morphologisch identisch zur klassischen HE‐Färbung (Abbildung 2).^57^ Dies kann sowohl bei der bioptischen Sicherung als auch zur Schnittrandkontrolle genutzt werden.

Aufgrund der kurzen Dauer bis zum feingeweblichen Bild stellt die *Ex‐vivo*‐KLM eine Alternative zur konventionellen (Kryostat‐)Schnellschnittdiagnostik dar. Im Gegensatz zum klassischen Schnellschnitt entsteht zudem bei der *Ex‐vivo*‐KLM kein Gewebeverlust. Die Sensitivität der Methode wird in mehreren Studien mit 73%‐100%, die Spezifität mit über 90% angegeben.^58^, ^59^, ^60^, ^61^

#### Plattenepithelkarzinom, Morbus Bowen und aktinische Keratosen

Die Darstellung von kutanen Plattenepithelkarzinomen mittels *Ex‐vivo*‐KLM mit digitaler Färbung ist ähnlich zum konventionellen HE‐Schnitt; die Tumorzellen erscheinen jedoch weniger eosinophil und Verhornungen führen zu einer stärkeren Reflektion. Die Kernfärbung mit Acridinorange erlaubt die Erkennung von Kernpleomorphien und atypischen Mitosefiguren. Ein invasives Wachstumsmuster ist gut zu erkennen.^55^ Aktinische Keratosen und der Morbus Bowen sind aufgrund der detaillierten Darstellung der Kernpleomorphien und atypischen Mitosefiguren morphologisch identisch zum HE‐Schnitt erkennbar und lassen sich vom Plattenepithelkarzinom gut unterscheiden.^62^

#### Melanozytäre Tumoren

Die Parameter zur Einordnung melanozytärer Tumoren wurden bereits 2017 analog der In‐vivo‐KLM definiert.^53^ Insgesamt erscheint die Beurteilung aufgrund der starken Reflektion des Melanins schwierig, sodass man nichtpigmentierte melanozytäre Naevi durchaus als solche erkennen kann,^49^ die Technik insgesamt jedoch (noch) nicht zur sicheren Differenzierung zwischen Melanomen und Naevi geeignet ist.

#### Entzündliche Dermatosen

Durch Anwendung der *pattern analysis* nach Ackermann^63^ ist mittels *Ex‐vivo*‐KLM eine orientierende Einschätzung entzündlicher Dermatosen innerhalb weniger Minuten möglich. Dazu wird die Anordnung des Entzündungszellinfiltrates in der Dermis (superfiziell, tief, perivaskulär, perifollikulär, interstitiell, lichenoid) oder im Fettgewebe (septale versus lobuläre Pannikulitis) beurteilt.^64^

Weitere Merkmale entzündlicher Dermatosen wie zum Beispiel die Spongiose oder die Akanthose lassen sich ebenfalls gut erkennen.^64^ Bei blasenbildenden Dermatosen kann zwischen intra‐ und subepidermaler Blasenbildung unterschieden werden. Perspektivisch kann die Technik auch für direkte Immunfluoreszenzuntersuchungen am Nativpräparat genutzt werden.^65^ Die so untersuchten Biopsien können ohne Gewebeverlust im Anschluss konventionell histologisch und immunhistochemisch aufgearbeitet werden.^63^

### Limitationen

Zu den aktuellen Herausforderungen zählen neben der Optimierung der Probenvorbereitung, Färbung und technischen Bilderzeugung, vor allem die korrekte Interpretation des *Ex‐vivo*‐Mikroskopiebildes.^66^

Die Bildgebung von großen Gewebestücken kann Schwierigkeiten bereiten, da das frisch exzidierte, nicht fixierte Gewebe eine maximale Präparatdicke von wenigen Millimetern nicht überschreiten sollte und oft einen makroskopischen, manuellen Zuschnitt erfordert.^67^

Durch Schwankungen in der Probendicke, ‐dichte und ‐beschaffenheit ist es zudem notwendig bei der Gewebemontage den ausgeübten Druck entsprechend möglichst gleichmäßig anzupassen, um eine gerade Gewebeoberfläche zu gewährleisten.^49^, ^51^

Unregelmäßigkeiten in der Gewebeoberfläche durch zum Beispiel Verunreinigung der Probe, unvollständigen Kontakt zum Objektträger durch Luftblasen sowie zu wenig Ultraschallgel als Medium für das Wasserimmersionsobjektiv können zu oben beschriebenen Artefakten und Einschränkungen der Bildinterpretation führen.^68^

Bei nicht darstellbaren Gewebeanteilen kann eine serielle Aufnahme in unterschiedlichen Eindringtiefen mit anschließender Fusion die Artefakte vermindern und eine automatische Mustererkennung mittels künstlicher Intelligenz (KI) ermöglichen.^69^

Bei der Untersuchung von Gefrierschnitten wird vermehrt eine unscharfe Färbung beobachtet, sodass die *Ex‐vivo*‐KLM an frischem Gewebe empfohlen wird.^70^

Zu den Einschränkungen beim Einsatz von KI in der *Ex‐vivo*‐KLM gehören technische Herausforderungen, wie nicht ausreichende Tumorerkennung durch die KI bei unscharfen Tumorrändern, ungleiche oder mangelhafte Kontrastierung insbesondere bei gleichzeitig auftretenden dichten Entzündungsinfiltraten. Zusätzlich sehen wir erhöhte Fehlerquoten bei der Unterscheidung zwischen Tumoranteilen und Hautadnexen. Eine suboptimale Abflachung des Gewebes vor dem Scannen bei der *Ex‐vivo*‐KLM führt zu vielen Artefakten und dadurch zu einer erschwerten Bewertung nicht nur für Experten, sondern auch für die KI.^71^

## OPTISCHE KOHÄRENZTOMOGRAPHIE (OCT)

### Technik

Bei der optischen Kohärenztomographie (OCT) werden Lichtstrahlen, meist einer Superlumineszenzdiode in das Gewebe gesendet^72^ und die Laufzeitdifferenzen der reflektierten Lichtanteile registriert. Die Kohärenzlänge der Lichtquelle legt die axiale Auflösung von 3–15 µm, die Linsenoptik die laterale Auflösung von bis zu 15 µm fest.^73^ Die Wellenlänge und indirekt die Lichtstreuung der Haut begrenzen die Eindringtiefe in die Haut auf 1–2 mm. Nach Verstärkung der Signalintensität können anhand einer logarithmischen Grauwert‐ oder Falschfarbenskala vertikale 2D‐Bilder erstellt werden.^73^, ^74^ Das strukturelle beziehungsweise konventionelle OCT‐Bild ähnelt histologischen Schnittbildern. Einige Geräte sind zudem mit einer dynamischen OCT‐Software ausgestattet und werden auch angiographische oder dynamische OCT (D‐OCT) genannt. Die D‐OCT basiert auf dem Prinzip der *speckle‐variance OCT*. Das bedeutet, die Software zeigt sich bewegende Teilchen als eine rote Überlagerung über das grau‐weiße strukturelle OCT‐Bild an.^75^

### Indikationen

#### Basalzellkarzinom

Neben dem hohen Stellenwert in der Diagnostik des Basalzellkarzinoms erlaubt das OCT durch die Visualisierung spezifischer epidermaler und dermaler Morphologien einen Rückschluss auf den zugrunde liegenden histologischen Subtyp: Noduläre Basalzellkarzinome präsentieren sich in der vertikalen und in der En‐Face‐Ansicht der strukturellen OCT‐Bildgebung als eine dermale hyporeflektive ovoide Struktur mit einer hyporeflektiven Spaltbildung und einem hyporeflektiven Randsaum (Abbildung 2).^76^ Die Nester finden sich immer in der Nähe von Haarfollikelstrukturen und sind typischerweise mit dem Haarschaft verbunden.^77^ Im hyperreflektiven kompakten Bindegewebssaum der Tumornester finden sich gestreckte, ovaläre und teils verzweigte Gefäße mit einem Durchmesser von bis zu 300 µm.^77^ Für superfizielle Basalzellkarzinome lassen sich hyporeflektive Nester oder ovoide Strukturen, von der Epidermis ausgehend, mit hyporeflektiven Vorwölbungen in die Dermis und epidermale Nester als wichtige Kriterien nennen.^76^ Die Gefäße erscheinen nicht verzweigt, dünn (< 40 µm), kurz (< 80 µm) und verlaufen locker spiralförmig im Bereich der DEJ.^77^ Für das sklerodermiforme Basalzellkarzinom ist eine traubenartige Erscheinung mit multiplen, von der Epidermis getrennten Knotenstrukturen oder kleineren aggregierenden Nestverbänden typisch.^76^ Die Tumornester sind typischerweise von dilatierten Gefäßen umgeben.^77^

#### Aktinische Keratosen und Plattenepithelkarzinom

Die OCT kann zudem ein geeignetes Verfahren in der Diagnostik des kutanen Plattenepithelkarzinoms und in der Differenzierung von Plattenepithelkarzinomen, aktinischen Keratosen und des Morbus Bowen sein.^78^ Aktinische Keratosen zeichnen sich durch morphologische Kriterien wie eine gestörte epidermale Zellschichtung, eine verdickte Epidermis und den Nachweis einer epidermalen hyperreflektiven Morphologie aus Streifen und Punkten aus.^79^ Bei Verdacht eines Plattenepithelkarzinoms sollte initial die Integrität der DEJ beurteilt werden. Kann diese nicht sicher und nur unvollständig nachvollzogen werden, liegt der Verdacht einer invasiven Infiltration durch ein Plattenepithelkarzinom nahe und erhärtet sich, falls eine hyperreflektive epidermale Tumorinfiltration mit Verwaschung der DEJ oder eine epitheliale periadnexale Infiltration vorliegt.^80^

#### Melanozytäre Läsionen (Nävi und Melanome)

Die strukturelle OCT besitzt in der Diagnostik von melanozytären Läsionen einen geringen Stellenwert, da eine eindeutige Diskriminierung zwischen Nävi und malignen Melanomen aufgrund der zu geringen Auflösung der strukturellen OCT nicht möglich ist.^81^, ^82^

Ein weiteres Kriterium zur Unterscheidung zwischen benignen und malignen melanozytären Läsionen ist die Beurteilung des Gefäßmusters. Welzel et al. konnten mithilfe der D‐OCT zeigen, dass sich bei Melanomen im Vergleich zur umgebenden gesunden Haut nicht nur vermehrte Blutgefäße darstellen lassen, sondern diese auch ein chaotisches Gefäßmuster aufweisen.^83^ Die Merkmale „atypisch geformte und irregulär verteilte Gefäße, erhöhte Gefäßdichte und erhöhter Gefäßdurchmesser“ waren signifikant mit Hochrisikomelanomen und metastasierten Melanomen assoziiert.^83^ Weiterhin konnte nachgewiesen werden, dass die Gefäßatypien positiv mit dem Breslow‐Index korrelieren.^84^

#### Inflammatorische und infektiöse Hauterkrankungen

Entzündliche und infektiöse Dermatosen, welche mit Veränderungen der Epidermis und der Durchblutung einhergehen, können mithilfe der (D‐)OCT untersucht werden. Im Gegensatz zur Beurteilung von nichtmelanozytärem Hautkrebs (NMSC) gibt es für die Untersuchung von den meisten inflammatorischen und infektiösen Hauterkrankungen bis dato wenig Evidenz und die Indikationen sind eher experimentell basiert.

### Limitationen

Die wichtigste Limitation der OCT ist die niedrige Auflösung, die auf Kosten der hohen Eindringtiefe physikalisch bedingt ist. Aufgrund der niedrigen Auflösung ist eine Darstellung von Einzelzellen nicht möglich. Außerdem können so melanozytäre Läsionen strukturell nicht unterschieden werden. Diese Einschränkung wurde mittlerweile durch die Weiterentwicklung der OCT zur *Line‐field* konfokalen optischen Kohärenztomographie (LC‐OCT) überwunden. Für eine optimale Messung ohne Bewegungsartefakte ist eine ruhige Positionierung des Patienten nötig, um Bewegungen des Patienten und des Untersuchers zu vermeiden. Dynamische Bewegungsartefakte äußern sich durch horizontale Linien. Am besten gelingt die Messung, wenn das OCT‐Gerät mit beiden Händen zur Messung stabilisiert wird. Zur OCT‐Untersuchung ist keine Vorbereitung der Haut mit Gel oder Öl nötig. Eine Messung ist daher schnell und dauert circa 30 Sekunden ohne dynamischen Modus und 60 Sekunden mit D‐OCT.

## *LINE‐FIELD* KONFOKALE OCT (LC‐OCT)

### Technik

Die *Line‐field* konfokale OCT (LC‐OCT) kombiniert die Grundlagen der OCT und KLM und erlaubt somit die hochauflösende (1–2 µm) Darstellung der Haut bis zur mittleren Dermis (circa 500 µm). Das Gerät besteht aus einem Zweistrahleninterferenzmikroskop, mit einer kontinuierlichen Laserquelle von 800 nm Wellenlänge und einer Zeilenkamera als Fotodetektor.

Im Detail basiert die LC‐OCT auf einem Time‐Domain‐OCT (TD‐OCT), wodurch multiple, parallele A‐Scans von der Hautoberfläche bis zu einer Tiefe von 500 µm für die Aufnahme von B‐Bildern hergestellt werden, während das Gerät ständig neu fokussiert. Die dynamische Live‐Fokussierung der B‐Scans erlaubt eine hohe Bildfrequenz, laterale (1,3 µm) und axiale (1,1 µm) Auflösung. Die Bilder sind schwarz‐grau‐weiß. Ihr Kontrast wird durch die unterschiedlich starke Reflektion der natürlichen Chromophore in der Haut wie Keratin und Melanin erzeugt. Der relativ hohe Brechungsindex im Vergleich zu Luft und Wasser stellt pigmentierte Zellen als sehr hell dar, im Gegenteil zum Beispiel zum wasserreichen, dunklen Zytoplasma. Die Aufnahmen werden in Echtzeit in drei Modi erstellt: vertikal (*en‐coupe*) wie bei der OCT und Histologie, horizontal (*en‐face*) wie bei der KLM und Dermatoskopie und 3D. Videoaufnahmen sind ebenfalls möglich. Die Navigation am Gerät wird durch eine auflichtmikroskopische Kamera gesteuert, um eine genaue Lokalisation des Scans zu erlauben.

Der Laser der Geräte entspricht der Laserklassifikation 1M nach EN 60825‐1, sodass es für die Anwendung ohne Sonderschutzausrüstung an Patienten, inklusive Kinder und Schwangeren, zugelassen ist.

### Indikationen

#### Basalzellkarzinome

Die LC‐OCT ermöglicht die morphologische Diagnose und Subtypisierung von Basalzellkarzinomen (BCC): Hierbei zeigen noduläre BCC atypische Keratinozyten, veränderte DEJ, Tumornester in der Dermis, hyporeflektive Spaltbildung, prominente Vaskularisation und weißes hyperreflektierendes Stroma, während oberflächliche Basalzellkarzinome eine Verdickung der Epidermis durch Tumorknoten mit Perlenkettenmuster und sklerodermiforme Basalzellkarzinome längliche hyporeflektierende Tumorstränge umgeben von hellem Kollagen (Fischschwarmmuster) zeigen (Abbildung 2).^85^, ^86^, ^87^

#### Feldkanzerisierung

Spezifische verschiedene Stadien keratinozytärer Tumoren wurden in zahlreichen Studien mit LC‐OCT untersucht. Dabei lag der Fokus auf der Morphologie der Keratinozyten sowie der Architektur von Epidermis und DEJ.^88^, ^89^, ^90^ Typische Merkmale, die mittels LC‐OCT darstellbar sind, umfassen unter anderem Hyperkeratose/Parakeratose, Unterbrechungen des Stratum corneum, eine verbreiterte Epidermis, basale und suprabasale Keratinozytenatypie, erweiterte Gefäße und Kollagenveränderungen. Während Plattenepithelkarzinome eine unterbrochene dermoepidermale Junktionszone sowie Ulzeration und Keratinpfropfen aufweisen, ist die DEJ bei aktinischen Keratosen und Morbus Bowen meist gut zu erkennen. LC‐OCT kann zudem basierend auf dem basalen Wachstumsmuster der Keratinozyten aktinische Keratosen klassifizieren und dabei die histologische PRO‐Klassifikation reproduzieren.^91^

#### Melanozytäre Läsionen

Mit der LC‐OCT besteht die Möglichkeit, melanozytäre Läsionen untersuchen zu können. Durch die hohe Auflösung können ähnlich der KLM einzelne Zellen analysiert werden, um eine Differenzierung zwischen Naevi und Melanomen potenziell zu erlauben.^92^, ^93^, ^94^, ^95^ Benigne Nävi zeigen wellenförmige Strukturen in der papillären und retikulären Dermis, die melanozytären Strängen/Nestern entsprechen (*wave pattern*).^92^ Für die Diagnose von Melanomen sind unregelmäßige wabenförmige Muster, pagetoides Wachstum von großen, runden, hyperrefraktiven Zellen in die Epidermis und das Fehlen von gut definierten, homogenen dermalen Nestern charakteristisch (Abbildung 1). Die LC‐OCT hat in einer Studie eine hohe Sensitivität und Spezifität für die Diagnose von Melanomen gezeigt, die Differenzierung von dysplastischen Nävi ist jedoch noch unklar.^93^

#### Entzündliche Dermatosen

Dank ihrer fast zellulären Auflösung ermöglicht die LC‐OCT eine Darstellung der Epidermis, DEJ und Dermis bis zu einer Tiefe von 500 µm. Bei entzündlichen Dermatosen kann sie potenziell eine In‐vivo‐Histopathologie‐Korrelation ermöglichen. Unter den am besten untersuchten Hauterkrankungen finden sich die bullösen Autoimmundermatosen, das Kontaktekzem und die Psoriasis, auch wenn die bisherigen Arbeiten als rein präliminär zu interpretieren sind. Die charakteristischen Merkmale wie Spongiose und Vesikelbildung lassen sich darstellen. Entzündliche Zellen erscheinen als refraktile Elemente, eine Subtypisierung ist jedoch in der Regel nicht möglich. Die Lokalisation der Spaltbildung kann hingegen intuitiv erfasst werden und lässt sich bei entsprechendem klinischem Verdacht erfolgreich für die nichtinvasive Diagnostik von Pemphigus foliaceus, Pemphigus vulgaris und bullösem Pemphigoid nutzen,^96^ anekdotisch auch bei pustulösen Dermatosen.^97^ Die Plaque‐Psoriasis zeigt sich in der LC‐OCT durch eine Verdickung des Stratum corneum und der Epidermis, elongierte Reteleisten sowie hyporefraktive, verlängerte dermale Papillen. Seltener sind Munro‐Mikroabszesse in Form subkornealer Konglomerate aus hyperrefraktiven Zellen darstellbar. Bei Ekzemen dominieren hingegen ein verdicktes und zerrissenes Stratum corneum mit abwechselnden hypo‐ und hyperrefraktiven Schichten sowie Spongiose und Vesikelbildung.^98^ Systematische Studien zu diesen Themen liegen bislang nicht vor, weshalb die diagnostischen Kriterien weiter definiert und standardisiert werden müssen.

### Limitationen

Die LC‐OCT ist zwar sehr intuitiv im Vergleich beispielweise zur KLM, erfordert allerdings detaillierte Kenntnisse der Histologie und Pathologie der Haut zur korrekten Interpretation der Bilder, so dass diese Technik in Form von Trainingskursen erlernt werden sollte. Die Auflösung ist nahezu zellulär, obwohl Einzelzellen manchmal schwer nosologisch zuzuordnen sind, insbesondere entzündliche Zellen. Die Eindringtiefe bis zur mittleren Dermis führt insbesondere bei dickeren Läsionen dazu, dass tiefere dermale Veränderungen – etwa bei nodulären Melanomen, knotigen Basalzellkarzinomen oder Pannikulitiden – nicht adäquat erfasst werden können. Die Methodik bleibt, wie in der Histologie oder Dermatoskopie anwenderabhängig.

## MULTIPHOTONENTOMOGRAPHIE

### Technik

Die Multiphotonentomographie (MPT) basiert auf dem Prinzip der Fluoreszenzanregung von endogenen Molekülen durch zwei oder mehr Photonen. Im Gegensatz zu Einphotonenmikroskopien, die Fluorophore durch relativ hohe und kurzwellige Energie anregen, beruht die Bildgebung der MPT auf Energieemissionen mittels langwelliger Strahlung im nahen Infrarotbereich.^99^

Durch autofluoreszierende Moleküle, wie Keratin, Melanin, Elastin, Porphyrine sowie freies und Protein‐gebundenes NADH, und das Phänomen der *Second Harmonic Generation* (SHG) werden epidermale und dermale Strukturen in der MPT in hochauflösender Form dargestellt. Die integrierte Darstellung der Fluoreszenzlebensdauern (Fluoreszenz Lifetime Imaging, FLIM) erlaubt zusätzlich die Analyse des zellulären metabolischen Zustandes und der molekularen Fingerabdrücke.^100^, ^101^ Die MPT produziert horizontale Schnittbilder – durch Rekonstruktion dieser Aufnahmen ist jedoch eine dreidimensionale Einschätzung des Gewebes möglich.^102^

Besonderer Vorteil der MPT ist die intravitale Analyse mit hoher Auflösung ohne Notwendigkeit einer vorherigen Färbung oder Markierung des Gewebes. Durch den Wegfall der damit verbundenen Artefaktbildung, wie zelluläre Schrumpfungsartefakte oder Flüssigkeitsinflux in den Interzellularraum, ist die MPT der konventionellen Histologie in dieser Hinsicht überlegen.

### Indikationen

#### Aktinische Keratose

Histopathologische Charakteristika der aktinischen Keratose in der MPT sind eine Akanthose, pleomorphe Keratinozyten, eine verschobene Kern‐Plasma‐Korrelation zugunsten der Nuclei sowie eine verminderte Zelldichte und vergrößerte, irreguläre Interzellularräume.^103^ Zellen der AK weisen heterogene Fluoreszenzmuster und Formen auf.^103^ Weiterhin kann ein vermehrter Kollagengehalt unterhalb der AK mit umgebender solarer Elastose der sonnenexponierten Haut dargestellt werden.^103^, ^104^, ^105^

#### Plattenepithelkarzinom

Plattenepithelkarzinome stellen sich in der MPT durch eine Verhornung in allen epidermalen Schichten dar.^103^ Fluoreszierende Zellkompartimente in Korneozyten und Keratinbündeln, die den histologischen Hornperlen des Plattenepithelkarzinoms entsprechen, lassen sich ebenso abbilden.^106^, ^107^ Die mikroskopischen Veränderungen der aktinischen Keratosen, wie das zunehmende Kern‐Plasma‐Verhältnis und die verminderte Zelldichte in den einzelnen epidermalen Schichten, sind im Plattenepithelkarzinom deutlich ausgeprägter verändert.^103^

#### Basalzellkarzinom

Das Basalzellkarzinom ist in der MPT charakterisiert durch einen Verlust der epidermalen Schichtordnung und monomorphe, elongierte Tumorzellen und Nuklei, die dicht gepackt und in eine oder doppelter Richtung polarisiert sind.^108^ Unter einer Exzitation von 760 nm lassen sich die Tumorzellnester durch umgebende parallel angeordnete Kollagen‐ und Elastinfasern darstellen. Dabei weisen die Tumorzellen eine längere Fluoreszenzlebensdauer auf als die umgebende Matrix.^109^ Bei einer Wellenlänge von 800–820 nm lassen sich die Tumorzellen nicht mehr abbilden, wodurch die extrazelluläre Matrix dunkle dermale Räume (*phantom islands*) zu umgeben scheint.^108^, ^109^

#### Melanom

Typische histopathologische Veränderungen des Melanoms, wie eingewanderte Melanozyten in der Epidermis und vergrößerte Interzellularräume mit unscharfer Abgrenzung zu den Melanozyten, sind in MPT‐Aufnahmen darstellbar.^110^ Zudem lassen sich pleomorphe, atypische Melanozyten, Zellfragmente sowie dendritische Zellen mit stark fluoreszierenden Dendriten in allen epidermalen Schichten abbilden.^110^, ^111^ Dimitrow et al. beschrieben die In‐vivo‐Diagnostik des Melanoms mittels der MPT mit einer Sensitivität von 75% und einer Spezifität von 80%.^110^ Die multiphotonentomographische Unterscheidung zu melanozytären Nävi wurde in einigen Studien untersucht. Im Gegensatz zum Melanom sind diese durch monomorphe und scharf begrenzte Zellen sowie eine reguläre Zellarchitektur charakterisiert.^112^ Ein signifikanter Unterschied der Fluoreszenzlebensdauern von melanozytären Zellen im Melanom und in melanozytären Nävi wurde nicht beobachtet.^113^

##### Entzündliche Dermatosen

##### Atopische Dermatitis

Die MPT kann die charakteristischen histologischen Merkmale der atopischen Dermatitis, wie die verdickte Epidermis und vergrößerte Interzellularräume durch entzündungsbedingte Ödeme, darstellen.^114^ Hier erweist sich die MPT im Vergleich zur klassischen Histologie aufgrund fehlender durch Einbettungs‐ und Färbungsverfahren verursachter Artefakte im Vorteil. Neben diesen pathologischen Veränderungen zeigt sich unter der MPT eine Reorganisation der Mitochondrien mit perinukleärer Akkumulation dieser.^114^, ^115^, ^116^ Durch Bestimmung des Verhältnisses der Fluoreszenzlebensdauer des freien und des Protein‐gebundenen NADH lässt sich die sogenannte mittlere Fluoreszenzlebensdauer berechnen, die den zellulären metabolischen Status entspricht.^117^, ^118^, ^119^ Huck et al. haben im Stratum granulosum von Patienten mit atopischer Dermatitis eine verminderte tau_m_ nachgewiesen, die mit dem Schweregrad der atopischen Dermatitis korreliert. Sie konnten ebenfalls eine verminderte tau_m_ in nichtläsionaler Haut von Patienten mit atopischer Dermatitis nachweisen, deren subklinischer entzündlicher Metabolismus in der klassischen Histologie meist okkult bleibt.^114^

##### Psoriasis vulgaris

Die Psoriasis vulgaris erscheint in der MPT mit einer Akanthose, Parakeratose und Munro‐Abszessen.^120^ Unter dem Stratum corneum psoriasiformer Plaques zeigt sich zudem ein charakteristisches, punktförmiges Fluoreszenzmuster, das wahrscheinlich durch die Parakeratose der Psoriasis hervorgerufen wird.^121^ Elongierte und dilatierte Papillen wurden unter der MPT beschrieben.^121^ Zurauskas et al. untersuchten mit der MPT die Fluoreszenzabklingzeiten von Psoriasis‐Patienten und konnten eine Korrelation mit dem *Psoriasis Area and Severity Index* (PASI) nachweisen.^122^ Ermöglicht wurde dies durch eine vollautomatische Analyse der optischen Biopsien.

### Limitationen

Grundsätzlich erfordert die MPT ein weitreichendes histopathologisches Wissen. Die horizontale Darstellung der Schnittbilder erschwert meist die Bildinterpretation. Eine weitere Limitation ist die geringe Eindringtiefe, die bei etwa 200 µm liegt.^101^ Stark gefältelte, eingesunkene, gewölbte, hyperkeratotische oder nässende Läsionen sind nur eingeschränkt beurteilbar. Aufgrund der hochauflösenden Aufnahmen im subzellulären Bereich sollten Bewegungsartefakte so gering wie möglich gehalten werden.

Eine zusätzliche Limitation der MPT sind die hohen Kosten, die durch die Anschaffung und möglichen Wartungsarbeiten einhergehen. Für die Messungen ist ein abgedunkelter und Temperatur‐kontrollierter Raum erforderlich. Ältere MPT‐Systeme sind durch ihre Größe und den bewegungseingeschränkten, optischen Arm limitiert. Neuere Generationen der MPT ermöglichen durch das kompaktere und kühlungsfreie System sowie den mobileren Arm eine praktikable Nutzung für den klinischen Alltag.^123^, ^124^

## WEITERE TECHNIKEN

### Optoakustik

Bei der optoakustische Bildgebung werden ultrakurze Lichtimpulse im sichtbaren bis infraroten Bereich von entsprechenden Biomolekülen im Gewebe (zum Beispiel Melanin, Hämoglobin, Lipide, Proteine, Wasser) absorbiert und erzeugen durch lokale thermoelastische Expansion Ultraschallwellen (photoakustischer Effekt). Algorithmen übersetzen die detektierten Schallwellen anschließend in eine dreidimensionale Darstellung, makroskopischer, mesoskopischer beziehungsweise mikroskopischer Auflösung.^125^, ^126^, ^127^

#### Multispektrale optoakustische Tomographie (MSOT)

Die multispektrale optoakustische Tomographie (MSOT) weist eine hohe Eindringtiefe mit guter Auflösung auf (Eindringtiefe > 10 mm, Auflösung 100–500 µm lateral und 100–500 µm axial).^128^ MSOT konnte bereits in verschiedenen dermatologischen Bereichen wie bei der Detektion von Melanommetastasen in Sentinel Lymphknoten (SLN), bei der Größenbestimmung (nichtmelanozytärer) Hauttumore oder bei der Bestimmung der Entzündungsaktivität bei Psoriasisarthritis vielversprechende Ergebnisse liefern. Für Forschungszwecke existieren bereits CE‐zertifizierte kommerziell erwerbbare Geräte.

#### Optoakustische Rasterscan‐Mesoskopie (RSOM)

Die optoakustische Rasterscan‐Mesoskopie (RSOM) vereint die Vorteile des tief eindringenden MSOT und der hochauflösenden optoakustischen Mikroskopie (OAM) und bietet ein besonders gutes Verhältnis von Eindringtiefe und Bildauflösung (Eindringtiefe 0–10 mm, Auflösung < 100 µm lateral und 10–100 µm axial).^128^ Nicht nur im Bereich der chronisch‐entzündlichen Dermatosen, sondern auch der melanozytären Neoplasien kann die RSOM die nichtinvasive Diagnostik und mikroskopisch kontrollierte Chirurgie ergänzen.^129^, ^130^, ^131^, ^132^

Limitierende Aspekte der RSOM‐Bildgebung sind unter anderem die lange Bildaufnahmezeit, die Empfindlichkeit gegenüber Bewegung (zum Beispiel durch Atmung) sowie eine geringe Eindringtiefe, welche die Bildqualität in der Tiefe einschränkt. Die Eindringtiefe und dementsprechend die Bildqualität ist zudem bei Fitzpatrick‐Hauttypen V und VI durch die starke Absorption durch Melanin eingeschränkt. Auch erschweren die Gerätedimensionen sowie ‐flexibilität Messungen an schwer zugänglichen, unebenen Körperstellen oder bei schwer beweglichen Patienten.^126^, ^133^

#### Die optoakustische Mikroskopie

Die optoakustische Mikroskopie bietet die höchste Auflösung unter den drei genannten Verfahren, geht jedoch mit geringer Eindringtiefe einher (Eindringtiefe 1–2 mm, Auflösung < 50 µm lateral und < 30 µm axial).^128^ Bisher findet die OAM momentan nur in experimentellen Studien Anwendung. In anderen Fachdisziplinen (unter anderem Augenheilkunde, Kardiologie, Gastroenterologie und Gynäkologie) wurde die Anwendung der OAM in präklinischen Studien untersucht.^134^, ^135^, ^136^

### Multispektralanalyse

Die Multispektralanalyse erfasst Gewebereflexionsdaten sichtbarer bis infraroter Wellenlänge und ermöglicht die präzise Quantifizierung und Analyse der spektralen, kolorimetrischen und räumlichen Merkmale der Hautbestandteile.

Die Multispektralanalyse kann als sensitive, vollautomatisierte, nichtinvasive und anwenderfreundliche Bildgebungsmethode ein mögliches zusätzliches Screeninginstrument darstellen. Auf Grund der geringen Spezifität und nutzerabhängigen Bildqualität findet die Multispektralanalyse aktuell noch keine routinemäßige Anwendung im klinischen Alltag.^137^, ^138^, ^139^, ^140^

### Raman‐Spektroskopie

Die Raman‐Spektroskopie untersucht die inelastische Streuung von Licht an Molekülen und Festkörpern. Die selektive, molekulare Darstellung von Hautstrukturen oder Substanzen wird wissenschaftlich zur Diagnostik von Hautläsionen (vor allem Melanomen) und der quantitativen Analyse von dermalem Wassergehalt, Photoaging, topischer Pharmakokinetik, Kosmetik und der Visualisierung von Tätowierungspigmenten angewandt.^141^, ^142^, ^143^, ^144^

Limitierende Faktoren für die routinemäßige klinische Anwendung und Verwendung in der Forschung sind die lange Bildaufnahmezeit, eingeschränkte Bildqualität und die komplexe Technologie verbunden mit relativ hohen Kosten.^144^

### Mikroelektrische Impendanzspektroskopie (MIS)

Die mikroelektrische Impendanzspektroskopie (MIS) ist kein optisches Verfahren, sondern misst den gewebsspezifischen Widerstand (Impedanz) und kann hieraus einen in Studien evaluierten elektrischen Impedanz‐Score (EIS 0–10) errechnen. Dieser Score korreliert mit bestimmten Wahrscheinlichkeiten der Malignität von keratinozytären Läsionen oder melanozytären Läsionen. In der Anwendung bei nichtmelanozytären Läsionen sind insbesondere seborrhoische Keratosen anderweitig klinisch auszuschließen, da sonst unnötig viele falsch positive, erhöhte EIS produziert werden. Werden seborrhoische Keratosen und entzündliche Läsionen klinisch oder dermatoskopisch ausgeschlossen, kann die MS eine wertvolle Entscheidungshilfe im Alltag sein.

### Laser‐induzierte Plasma‐Spektroskopie (LIPS)

Ein neues Verfahren zur Dignitätsanalyse suspekter Läsionen ist die Laser‐induzierte Plasma‐Spektroskopie (LIPS). Ähnlich wie bei der MIS erfolgt die Ergebniseinschätzung über einen Score, der beim weiteren Management der Läsion hilfreich sein kann. Hierbei wird mit einem Nanosekunden Neodym‐YAG Laser Impuls aus der Läsion ein Mikroplasma erzeugt, welches dann spektroskopisch über eine KI‐Software analysiert wird. Die Technik ist auf der Haut schmerzfrei und nichtinvasiv. Pro Läsion werden drei Messungen durchgeführt. In anderen medizinischen und physikalischen Bereichen wird die LIPS bereits als schnelles und genaues Werkzeug eingesetzt (Leberkarzinome, kolorektale Karzinome, Brustkrebs). Die Plasma Veränderungen (unteranderem Calcium‐ Zink‐ und Kupfer‐Konzentration) korrelieren zu Zellproliferation, ‐apoptose und ‐differenzierung.^145^

## METHODENVERGLEICH IN EINER ÜBERSICHT

Tabelle 1 gibt eine Übersicht der dargestellten Bildgebenden Technologien samt technischer Spezifikationen, Hauptindikationen und Gerätekosten.

**TABELLE 1:** Übersicht innovativer, nichtinvasiver bildgebender Technologien für die dermatologische Diagnostik.

	Auflösung	Eindringtiefe	Messdauer	Bildausschnitt (horizontal, vertikal, 3D)	Bildinterpretation	Gerätekosten	Hauptindikation (melanozytär, NMSC, inflammatorisch)
In‐vivo‐KLM	1–3 µm	Bis zu 250 µm	2–5 min	Horizontal	Detaillierte Kenntnisse der Dermatopathologie erforderlich; Trainingskurse	Ab 70 000–185 000 € (je nach Ausstattung)	Epitheliale und vorrangig melanozytäre Tumoren mit höherer Auflösung
*Ex‐vivo*‐KLM	1–3 µm	Bis zu 250 µm	< 1–5 min (abhängig von Gewebsgröße)	Horizontal und vertikal	Detaillierte Kenntnisse der Dermatopathologie erforderlich	Ab 249 000 €	Schnellschnittuntersuchung von Frischgewebe von epithelialen und melanozytären Tumoren; weitere Disziplinen (u. a. Gynäkologie und Urologie)
OCT	10–15 µm	Bis zu 1,5 mm	0,5–1 min	Horizontal und vertikal	Detaillierte Kenntnisse der Dermatopathologie erforderlich	Ab 85 000 €	Epitheliale Tumoren mit höherer Eindringtiefe
LC‐OCT	1–3 µm	Bis ca. 500 µm	2 min	Horizontal, vertikal, 3D und Videoaufnahmen	Detaillierte Kenntnisse der Dermatopathologie erforderlich; Trainingskurse	Ab 150 000 €	Epitheliale und melanozytäre Tumoren mit höherer Auflösung
MPT	0,5–2 µm	Bis zu 200 µm	5–15 min	Horizontal	Detaillierte Kenntnisse der Dermatopathologie erforderlich; Trainingskurse für Interpretation notwendig	Ab 290 000‐325 000 € (je nach Ausstattung)	Inflammatorische Hauterkrankungen (V. a. atopische Dermatitis und Psoriasis), akute und chronische Wunden, epitheliale/melanozytäre Tumoren
Optoakustik	Je nach Methodik < 1 µm bis 100–500 µm lateral und axial	Über 10 mm	1–15 min	3D	Detaillierte Kenntnisse der Dermatopathologie erforderlich; technische Trainingskurse erforderlich; anwenderabhängig	MSOT: 350 000‐500 000 € RSOM: 130 000‐150 000 €	Melanom‐Metastasen in Sentinel‐Lymphknoten; Größenbestimmung von Hauttumoren; Entzündungsaktivität und Therapiemonitoring bei inflammatorischen Erkrankungen
Multispektral‐analyse	20 µm	Bis zu 2,5 mm	3 min	Horizontal	Computer‐assistierte Bildverarbeitung ermöglicht Klassifizierung von melanozytären Läsionen	Momentan kommerziell nicht erhältlich.	Zusätzliches Screeningtool (noch keine routinemäßige Anwendung im Alltag)
Raman‐spektroskopie	Abhängig von Methodik bis zu wenigen µm	Abhängig von Wellenlänge bis zu mehreren hundert µm	3–10 min	Ramanspektrum	Spektralanalyse mit Vergleich zu Referenzspektren; anwenderabhängig; Trainingskurse erforderlich	Momentan kommerziell nicht erhältlich	Epitheliale/melanozytäre Tumoren
***Nicht‐optische Verfahren***				***Ausgabemodus***			
Mikroelektrische Impedanz‐spektroskopie	–	Bis in obere Dermis	3–5 min	EIS	Impedanzanalyse im Vergleich zu normalem Gewebe	Ab 6900 €	Epitheliale/melanozytäre Tumoren
Laser‐induzierte Plasma‐spektroskopie	–	Epidermale Läsionen	3–5 min	LIPS‐Score	Plasmaspektralanalyse im Vergleich zu normalem Gewebe	Ab 42 000 €	Epitheliale/melanozytäre Tumoren

*Abk*.: In‐vivo‐KLM, In‐vivo‐konfokale Laserscanmikroskopie; *Ex‐vivo*‐KLM, *Ex‐vivo*‐konfokale Laserscanmikroskopie; OCT, Optische Kohärenztomographie; LC‐OCT, *Line‐field* konfokale Optische Kohärenztomographie; MPT, Multiphotonentomographie; 3D, dreidimensional; EIS, Elektrischer Impedanzscore; LIPS, Laser‐induzierte Plasmaspektroskopie

## DANKSAGUNG

Open access Veröffentlichung ermöglicht und organisiert durch Projekt DEAL.

## INTERESSENKONFLIKT

Siehe Langfassung der Leitlinie unter www.awmf.org.
